# Human colon cancer profiles show differential microRNA expression depending on mismatch repair status and are characteristic of undifferentiated proliferative states

**DOI:** 10.1186/1471-2407-9-401

**Published:** 2009-11-18

**Authors:** Aaron L Sarver, Amy J French, Pedro M Borralho, Venugopal Thayanithy, Ann L Oberg, Kevin AT Silverstein, Bruce W Morlan, Shaun M Riska, Lisa A Boardman, Julie M Cunningham, Subbaya Subramanian, Liang Wang, Tom C Smyrk, Cecilia MP Rodrigues, Stephen N Thibodeau, Clifford J Steer

**Affiliations:** 1 Biostatistics and Informatics, Masonic Cancer Center, University of Minnesota, Minneapolis, Minnesota, USA; 2 Department of Laboratory Medicine and Pathology, Mayo Clinic, Rochester, Minnesota, USA; 3 Research Institute for Medicines and Pharmaceutical Sciences, Faculty of Pharmacy, University of Lisbon, Lisbon, Portugal; 4 Department of Laboratory Medicine and Pathology, University of Minnesota, Minneapolis, Minnesota, USA; 5 Department of Health Sciences Research, Division of Biomedical Informatics and Statistics, Mayo Clinic, Rochester, Minnesota, USA; 6 Division of Gastroenterology and Hepatology, Mayo Clinic, Rochester, Minnesota, USA; 7 Department of Medicine, University of Minnesota, Minneapolis, Minnesota, USA; 8 Genetics, Cell Biology and Development, University of Minnesota, Minneapolis, Minnesota, USA

## Abstract

**Background:**

Colon cancer arises from the accumulation of multiple genetic and epigenetic alterations to normal colonic tissue. microRNAs (miRNAs) are small, non-coding regulatory RNAs that post-transcriptionally regulate gene expression. Differential miRNA expression in cancer versus normal tissue is a common event and may be pivotal for tumor onset and progression.

**Methods:**

To identify miRNAs that are differentially expressed in tumors and tumor subtypes, we carried out highly sensitive expression profiling of 735 miRNAs on samples obtained from a statistically powerful set of tumors (n = 80) and normal colon tissue (n = 28) and validated a subset of this data by qRT-PCR.

**Results:**

Tumor specimens showed highly significant and large fold change differential expression of the levels of 39 miRNAs including miR-135b, miR-96, miR-182, miR-183, miR-1, and miR-133a, relative to normal colon tissue. Significant differences were also seen in 6 miRNAs including miR-31 and miR-592, in the direct comparison of tumors that were deficient or proficient for mismatch repair. Examination of the genomic regions containing differentially expressed miRNAs revealed that they were also differentially methylated in colon cancer at a far greater rate than would be expected by chance. A network of interactions between these miRNAs and genes associated with colon cancer provided evidence for the role of these miRNAs as oncogenes by attenuation of tumor suppressor genes.

**Conclusion:**

Colon tumors show differential expression of miRNAs depending on mismatch repair status. miRNA expression in colon tumors has an epigenetic component and altered expression that may reflect a reversion to regulatory programs characteristic of undifferentiated proliferative developmental states.

## Background

Colon cancer (CC) is the second most frequent cause of cancer-related death in the United States and in Europe [1,2]. The development of CC is considered a stepwise process with the accumulation of different genetic and epigenetic alterations. Most tumors (~85%) are generated by chromosomal instability (CIN) and associated with high frequency aneuploidy and allelic imbalance. The remaining 15% have defective DNA mismatch repair (dMMR), which is frequently measured by either the presence of microsatellite instability (MSI) or by testing for loss of the protein products for genes involved in DNA mismatch repair, most commonly *MLHI, MSH2, MSH6*, and *PMS2 *[3]. Sporadic CCs with dMMR have distinctive clinical and pathological features that include proximal colon predominance, poor differentiation and/or mucinous histology, intra- and peritumoral lymphocytic infiltration, diploid DNA content [3] and generally have a better prognosis [4]. The presence of MSI-H tumor phenotype and loss of protein expression of *MLHl *and *MSH2 *is highly concordant [5].

Recent progress has been made in CC screening [6] and treatment protocols [7]. Currently, the most important prognostic factor for patients is pathologic tumor staging based on the tumor-node-metastasis (TNM) system [8]. However, several pathologic and clinical features have been associated with increased risk of tumor recurrence in resectable CC [7]. Between 20 and 25% of stage II CC patients develop recurrence and die from the disease [9]. Therefore, it is imperative to identify and develop accurate, reliable and sensitive biomarkers.

Genome-wide approaches have reshaped the landscape of cancer research. Meta-analysis of multi-study data has allowed the identification of overlapping sets of differentially expressed genes that may have biomarker potential [10]. Emphasis on genome-wide gene expression analyses has been driven by the general view that alterations in protein-coding oncogenes or tumor-suppressor genes underlie tumorigenesis. However, the discovery of a growing class of small non-coding RNAs, termed miRNAs, has opened a new field of cancer research and revealed the complexity of cancer biology. miRNAs regulate gene expression post-transcriptionally by translational attenuation/repression or cleavage of target mRNAs [11], thereby adding a new dimension to the regulation of gene expression [12]. Further, aberrant expression of miRNAs has been associated with a growing list of cancers [13].

The potential use of miRNAs in diagnosis and prognosis has been demonstrated for several forms of cancer [14,15]. miRNA expression profiles may be better-suited targets for the discovery of novel cancer biomarkers compared to gene expression profiles. This is supported by a report demonstrating the ability of miRNA profiles to correctly classify human cancers of unknown primary origin as well as poorly differentiated tumors [16]. A growing number of studies have addressed miRNA expression in CC [17-20]. However, comparison across studies is limited by differences in profiling platform, quantity of miRNA obtainable, methodology, in some cases sample number and a paucity of clinicopathologic data. Consequently, translation of results into clinically useful and widely applicable biomarkers is hampered. Importantly, a potentially strong contributor to the variability of data among different studies relates to the tumor resection procedures. The inadvertent collection of surrounding residual non-tumor tissue may skew experimental results, diluting quantitative estimates of particular miRNA species based on the extent and type of tissue present in the sample. Most studies do not specifically address this potential problem.

In this study, global miRNA expression was evaluated in 80 colon tumors and 28 normal mucosa samples using the BeadArray™ platform (Illumina, Inc.) to evaluate global miRNA expression of 735 miRNA targets [21]. Our results demonstrate that, in a sufficiently statistically powered number of tumors, a larger set of miRNAs than has previously been reported is differentially expressed between normal colon and tumor tissue. Additionally, specific miRNAs are significantly differentially expressed between tumors of deficient and proficient mismatch repair status (dMMR and pMMR, respectively).

## Methods

### Colon Cancer Samples

CC biospecimens were obtained from Mayo Clinic Rochester patients with colorectal neoplasia beginning in 1995. Of the consented patients, US residents who had only CC were included in this study. The majority of patients in the registry are from the surrounding five-state region. Pathologic tumor staging was carried out using the tumor-node-metastasis (TNM) system[8]. Collection of tissue material and review of patient records to obtain clinical information were performed under IRB-approved protocol.

### Mismatch Repair Status

Defective DNA mismatch repair (dMMR) was defined by the presence of microsatellite instability (MSI-H) and/or the absence of protein expression for *hMLH1*. Proficient DNA mismatch repair (pMMR) was defined by the absence of microsatellite instability (MSS/MSI-L) and the presence of normal protein expression for *hMLH1*. MSI in CC cases was compared with paired normal and tumor DNA isolated (Qiagen DNA extraction kit) from formalin-fixed, paraffin-embedded (FFPE) material. Tumors were classified as MSI-H if > 30% of markers demonstrated instability and as MSS/MSI-L if < 30% demonstrated MSI [22,23]. Immunohistochemical analysis of hMLH1 expression was performed on FFPE samples, as previously described [5].

### RNA Extraction

Following harvest, tissue samples were immediately snap frozen and stored at -80°C. For each case, frozen tumor tissue was cut on a cryostat to generate a hematoxylin and eosin (H&E) stained slide. Tumors were then evaluated for content of tumor present, areas of tumor containing at last 70% tumor or greater were macro-dissected and tissue equivalent to 7 mm^2 ^and 10-microns thick was then sectioned and placed in a vial containing 400 uL of RLT buffer (QIAGEN, Chatsworth, CA) including 4 μL of β-mercaptoethanol. The vial was then stored at -80°C until utilized for RNA extraction using TRIzol^® ^LSTrizol© (Invitrogen, Corp., Carlsbad, CA), according to the manufacturer's instructions.

### miRNA Expression by qRT-PCR

qRT-PCR was carried using normalization to U6 snRNA. First strand cDNA was synthesized from 1 μg of total RNA using a miScript reverse transcription kit (Qiagen, Valencia, CA). miRNA was quantified with an miRscript SYBR Green PCR kit (Catl: 218073, Qiagen, Valencia, CA) using cDNA equivalent of 50 ng total RNA per reaction. Mature miRNA-specific forward primers [see Additional file 1] were purchased from a vendor (IDT, USA) and the universal reverse primer provided by the manufacturer. Real-time PCR was performed at 55°C annealing following standard protocol of the manufacturer in 7500 Real Time PCR system (Applied Biosystem, USA) and threshold cycles (*C*_T_) were calculated using Sequence Detection Software (SDS v1.2.1, Applied Biosystem, USA). Fold expression was calculated from the triplicate of *C*_T _values following the 2^-ΔΔCt ^method [24].

### RNA Processing

The chemistry involved in the miRNA BeadArray™ was similar to that used in the DASL amplification process [21]. Total RNA [200 ng (all 108 samples) and 400 ng (38 technical replicates)] was poly-adenylated and then converted to cDNA using a biotinylated oligo-dT primer with a universal PCR sequenced at its 5'-end. This was followed by annealing of a miRNA-specific oligonucleotide pool (MSO), which consisted of three parts: a universal PCR priming site at the 5' end; an address sequence complementary to a capture sequence on the BeadArray™; and a microRNA-specific sequence at the 3'-end. Extension of MSO was facilitated by addition of a polymerase but only if their 3' bases were complementary to the cognate sequence in the cDNA template. Common primers were used to amplify the cDNA templates; the primer complimentary to the BeadArray™ was fluorescently labeled. The single-stranded PCR product was hybridized to the Sentrix Array Matrix where the labeled strand bound to the bead on the array containing the complementary address sequence. The arrays were imaged using an Illumina BeadArray™ Reader, which measures the fluorescence intensity at each addressed bead location. Intensity files were analyzed using BeadStudio version 3.1.1. Expression levels were expressed as an average Beta value.

### Pre-processing and Analyses

Data were exported from Illumina's BeadStudio package, with neither background correction nor scale correction, and transformed to the log base 2 scale. Quantile normalization [25] was used as implemented by the R library affy [26,27]. Samples were distributed on two Illumina SAMs; systematic effects due to SAM were evaluated and found to be minimal. A linear mixed-effects model [28] was used on a probe-by-probe basis to compare expression between groups accounting for the correlation between replicates or paired normal-tumor samples from a given subject. Replicate samples were averaged for further data analyses.

Support Vector Machine Classification (SVM) was carried out using the Genedata expressionist data analyses suite. Clustering was carried out on log transformed median centered data using Cluster 3.0 for Mac OS X (C Clustering Library 1.43) centroid linkage clustering and was visualized using Java TreeView Version 1.1.3. miRNA tumor expression data has been submitted to NCBI's Gene Expression Omnibus (GEO) as ID: GSE18392. Additionally, the data will be searchable on our website http://www.oncomir.umn.edu.

Genomewide CC methylation data [29], genes implicated in CC via forward genetic screen [30] and stem cell miRNA expression data [31] were obtained from publication. The Diana miRpath application [32] was used to determine pathway enrichment within the putative targets for miRNAs found to be altered in CC. miRNA::mRNA interactions were downloaded from the miRDB version 2.0 database. Custom Perl scripts were used to parse miRDB, identify predicted miRNA interactions with tumor suppressor mRNA and identify relationships between miRNA and methylated regions.

## Results

### Tissue Specimens

To systematically determine differences in miRNA expression in colon cancers and their subgroups from normal colon tissue, the expression levels of 735 miRNA in 108 tissue samples were examined using the Illumina miRNA detection platform. The characteristics of these tissue samples are described in Table 1 with additional data provided [see Additional file 2]. All tumors were histologically reviewed and macrodissected to ensure that at minimum 70% of the tumors contained neoplastic cells to ensure that the majority of tissue extracted was of neoplastic origin. The colon tumors were further separated by mismatch repair status into dMMR (n = 12) of acquired variety (sporadic) and pMMR groups (n = 68). The remaining 28 tissue specimens were derived from adjacent normal colon tissue removed during tumor resection. All data, both raw and normalized, is provided [see Additional file 3].

**Table 1:** Sample counts of tissues meeting specified characteristics analyzed in this study

Characteristic	Count
normal colon	28
dMMR/MSIH tumors (sporadic)	12
pMMR Stage II tumor	28
pMMR Stage III tumor	24
pMMR Stage IV tumor	15
pMMR Stage undefined	1
**Total Profiled**	**108**

### Technical Array Replicates Show High Level of Correlation

**Figure 1:**
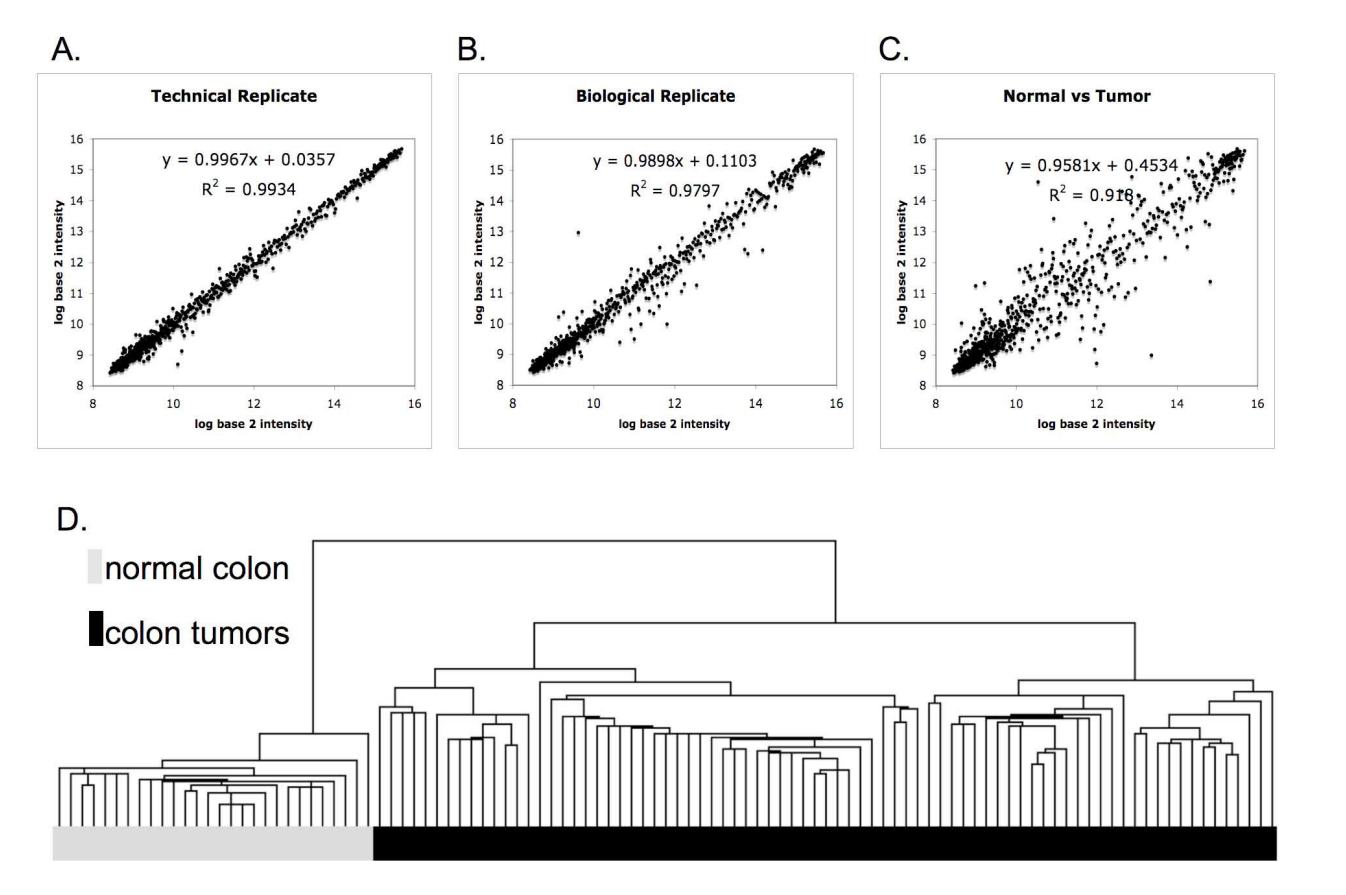
**Intensity plots and unsupervised clustering of miRNA profiles**. (A-C) Log base 2 intensity plots between representative samples. The equation and Pearson Correlation r values establish the linear fit of data. Comparison of array data obtained from (A) a technical replicate - tumor#101 obtained using 200 ng total RNA (x-axis) with the data obtained using 400 ng total RNA (y-axis; r = 0.997); (B) a biological replicate - normal#185A (x-axis) with normal#385A (y-axis; r = 0.990); and (C) a normal tumor comparison - normal#171A (x-axis) with tumor#334 (y-axis; r = 0.918). (D) Unsupervised hierarchical clustering of 108 miRNA profiles using 735 miRNA.

Representative log base 2 intensity plots for direct comparison of normalized array data are shown for pairs of technical replicates, biological replicates and Normal/Tumor (Figure 1). Technical replicates using either 200 ng or 400 ng of RNA were used to determine the reproducibility of the Illumina miRNA platform. High Pearson correlation r values were found between technical replicates even when using different concentrations of RNA for amplification (Average 0.993; Range {0.977 to 0.997}) (Figure 1A). Lower, but still high, correlations were seen between biological replicates Normal to Normal (Average 0.984; Range {0.951 to 0.996}) (Figure 1B). The lowest correlations were seen between normal colon tissue and tumor tissue [Average 0.967; Range {0.891 to 0.985} (Figure 1C)].

### miRNA Profiles Show Distinct Separation of Tumors from Normal Tissue

Unsupervised hierarchical clustering was carried out on the miRNA expression data (Figure 1D). Analyses of the dendrogram revealed that normal colon tissue profiles cluster together in a group separate from the colon tumor samples (Figure 1D). In addition, normal tissue gave a tighter internal correlation, signified by higher correlations, than the tumor tissue subset, which as a group showed less internal correlation.

SVM analyses were used to determine whether the miRNA profiled by the unsupervised hierarchical clustering are sufficient to classify the tumor/normal status. SVMs look for distinctive patterns in groups of data and then use them to classify group membership status for additional data. A Monte Carlo cross-validation simulation was set up to create training sets from 50% of the data and use the sets to predict tumor/normal status of the remainder of the dataset with 1000 iterations. Following combination of the results from all simulations, tumor vs normal status was correctly classified 100% of the time. The confusion matrix is included [see Additional file 4].

### Differential Expression

**Figure 2:**
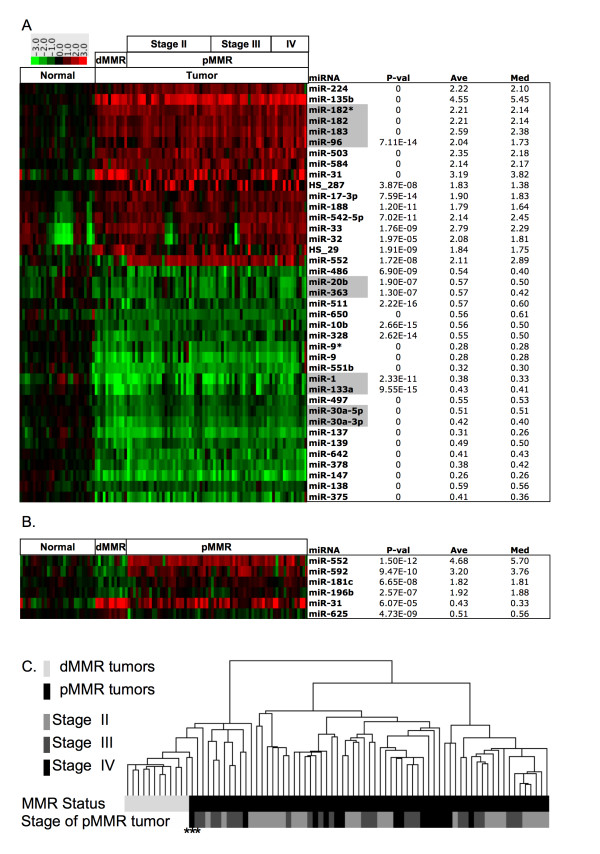
**Differentially expressed miRNA between normal colon and tumor tissue**. Heatmaps of significantly differentially expressed miRNAs. All miRNA expression data is shown relative to the average value of normal colon. Both tissue median and average fold-change values between the two comparison groups are shown. miRNAs found in close proximity within the genome that also show correlation with each other are highlighted in gray. P-values < 10e-16 represented as 0. (A) Normal colon samples compared to tumors. (B) dMMR colon cancers are compared to pMMR colon tumors. (C) Unsupervised hierarchical clustering of tumors using 43 miRNA found to be significant. Samples improperly defined by SVM cross-validation denoted with asterisk.

A linear mixed effects model was used to determine statistically significant miRNAs differentially expressed in normal tissue compared with all tumor tissues (Figure 2; detailed statistics also provided [see Additional file 5]). In order for a miRNA to be rigorously considered significant, a *p <*6.8e-5 was required. This represents the Bonferroni correction for solving the multiple testing problems associated with carrying out 735 individual tests, one for each miRNA studied on the array. In addition, significantly altered miRNAs were also required to show a fold change of absolute value greater than 1.7 to further focus on miRNA with large expression changes in our high tumor content samples.

Using these criteria, 39 miRNAs were differentially expressed between normal colon tissue and the composite collection of colon tumor tissue (Figure 2A). Many of these miRNA show similar expression patterns and are found in close chromosomal proximity. A subset of 17 miRNAs showed increased expression in colon tumors relative to normal colon tissue. Importantly, miR-135b displayed the largest average change, a 4.55-fold increase. miR-96, miR-182, miR-182* and miR-183 were all up-regulated; expression was highly correlated and mapped to the same region of chromosome 7. Additionally, 22 miRNAs showed decreased expression in colon tumors. Among the down-regulated miRNAs, miR-133a and miR-1, as well as miR-30a-3p and miR-30a-5p, have a common chromosomal locus on chromosome 6 and chromosome 18 respectively and also display highly correlated expression levels. Further, miR-20b and miR-363 show similar levels and are expressed from a common region on chromosome X.

### miRNAs Show Striking Differences with Mismatch Repair Status

Direct comparison of dMMR with pMMR tumors by linear effects mixed model using the Bonferroni correction revealed that six miRNAs were differentially expressed, with high significance, between different tumor subtypes (Figure 2B; detailed statistics provided [see Additional file 5]). miRNAs that showed decreased levels in pMMR relative to dMMR tumors included miR-552, miR-592, miR-181c and miR-196b. In contrast, miR-625 and miR-31 exhibited increased levels in dMMR relative to pMMR tumors.

As expected, unsupervised hierarchical clustering, (Figure 2C) as well as SVM analyses, were able to demonstrate separation of dMMR from pMMR tumors using statistically significant miRNAs as defined in Figure 2A-B. However, separation was not as complete as that observed between normal and tumor tissues using all probes (Figure 1D). Examination of the hierarchy generated revealed that a pMMR tumor subset gave expression patterns resembling those found in dMMR tumors, when analyzing statistically significant miRNAs. The area for which SVM was unable to correctly define the tumor MMR status was also the boundary region observed by unsupervised hierarchical clustering between dMMR and pMMR tumors. The SVM confusion matrix for this analysis is included [see Additional file 4]. Further, unsupervised clustering was not able to differentiate between pMMR stages II, III and IV using either the complete dataset or the statistically significant tumor dataset. Furthermore, no differences were observed between tumors from males and females.

### dMMR vs pMMR Significant miRNAs Show Differential Levels of Response Relative to Normal Colon Tissue

**Figure 3:**
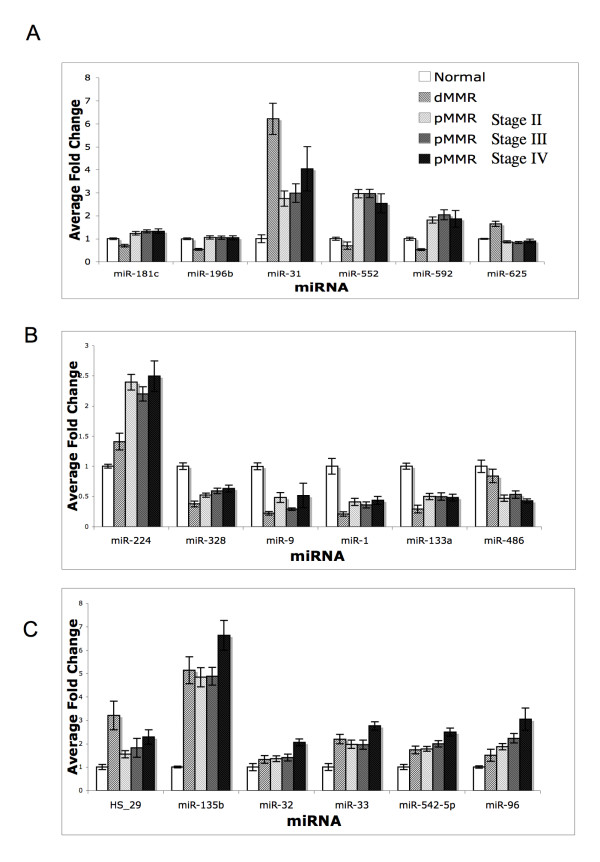
**Differentially expressed miRNA by tumor subtype**. Bar chart describing relative expression levels for each subgroup relative to the average expression level in normal tissues. Error bars represent the standard error. (A) miRNA with highly significant differences between dMMR and pMMR tumors. (B) Tumor significant miRNA with *p *< 0.05 and a fold change > 1.5 for the comparison of dMMR and pMMR tumors. (C) Tumor significant miRNAs with *p *< 0.05 and fold change > 1.5 for the comparison of pMMR stage II and pMMR stage IV tumors.

The average fold change of the significantly altered miRNAs found in the direct comparison between dMMR tumors and pMMR tumors was evaluated relative to normal tissue across all tumor subgroups (Figure 3A). Comparing these expression levels with those observed in normal colon tissue revealed the following patterns: miR-31 was significantly up-regulated in colon tumors relative to normal colon tissue and this increase was significantly greater in dMMR (7-fold) than in pMMR tumors (4-fold). In addition, miR-552 and miR-592 expression was up-regulated in pMMR tumors and down-regulated in dMMR tumors. miR-625 showed increased expression in dMMR tumors and unchanged in pMMR tumors, relative to normal tumors. In a directly opposite fashion, miR-196b and miR-181c show decreased levels of expression only in dMMR tumors.

Examination of miRNAs showing highly significant changes in tumor vs normal revealed that the expression levels for 6 miRNAs were different (*p *< 0.05 and fold change > 1.5) between dMMR and pMMR tumors (Figure 3B). miR-1, miR-133a, miR-328 and miR-9 are further decreased in dMMR tumors relative to pMMR tumors. miR-224 was induced slightly in dMMR and further increased in pMMR tumor. miR-486 was reduced only in dMMR tumors; and HS-29 was induced to a greater extent in dMMR tumors than pMMR tumors.

While the majority of miRNAs that were differentially expressed in pMMR tumors showed similar perturbations between stages, stage IV pMMR tumors displayed a trend towards increased levels of a subset of miRNAs, relative to stage II and stage III pMMR tumors. Six of the miRNAs identified as highly significant to tumors (HS-29, miR-135b, miR-32, miR-33, miR-542-5p and miR-96) displayed higher expression in pMMR stage IV as compared to stage II tumors (*p *< 0.05 and fold change > 1.5) (Figure 3C). The levels found in Stage III tumors trended towards those identified in Stage II tumors.

### Chromosomal Regions Containing Differentially Expressed miRNAs Also Show Alterations in DNA Methylation Status

Recently, 2,707 regions of differential DNA methylation were identified after analyzing ~4.6 million CpG sites in the human colon cancer genome [29]. Using a window size of 40,000 bp, statistically significant differentially expressed miRNAs identified in the current study localized to 28 specific chromosomal regions. Of these, 10 co-localized to regions differentially methylated in colon cancer as defined. Using Fisher's Exact test, the null hypothesis that these events were unrelated was rejected. Of the 28 regions containing miRNA differentially expressed in colon cancer, 10 were found in regions in which a maximum of 2,707 were differentially methylated in tumors, out of 75,000 potential windows (3,000,000,000 bases/40,000 base windows). The overlap between differential miRNA expression and methylation is much greater than what would be expected by random chance (*p *= 2.75e-8) (Table 2A). Of interest, the majority of these miRNA's (9/10) were repressed in colon tumors. Additionally, for 8 out of 10 regions, the closest differentially methylated region showed an increase in methylation (Table 2B).

**Table 2:** Fishers Exact test to determine probability of overlap between differentially expressed miRNAs and differentially methylated regions in colon cancer and the regions of overlap

A				
		Regions with Δ miRNA transcript		Genomic regions
**Regions with Δ methylation**		10		2707
**Regions with no Δ methylation**		18		72293
**p-value**		2.75e-8		

**B**				
**Chromosome**	**Location**	**Object**	**Distance (bp)**	**Δ Methylation**

X	133131074	miR-363		
X	133131505	miR-20b		
X	133135052	C-DMR	3547	0.45
**X**	**133507562**	**C-DMR**	**462**	**0.39**
**X**	**133508024**	**miR-503**		
**X**	**133508504**	**C-DMR**	**480**	**0.42**
1	98284214	miR-137		
1	98291025	C-DMR	6811	0.37
**15**	**87712252**	miR-9*		
**15**	**87712252**	**miR-9**		
**15**	**87745140**	**C-DMR**	**32888**	**0.39**
**15**	**87749926**	**C-DMR**		**0.37**
16	65755256	C-DMR	38469	0.47
16	65793725	miR-328		
**2**	**176711496**	**C-DMR**	**11781**	**0.37**
**2**	**176723277**	**miR-10b**		
2	219556012	C-DMR	18599	0.53
2	219574611	miR-375		
**22**	**21495270**	**miR-650**		
**22**	**21526783**	**C-DMR**	**31513**	**-0.56**
6	72169975	miR-30a-5p		
6	72169975	miR-30a-3p		
6	72185916	C-DMR	15941	0.38
6	72187453	C-DMR		
**8**	**41637116**	**miR-486**		
**8**	**41676418**	**C-DMR**	**39302**	**-0.5**

Genomic regions are grouped by alternating bold font. Figures

### Validation of miRNA Expression

**Figure 4:**
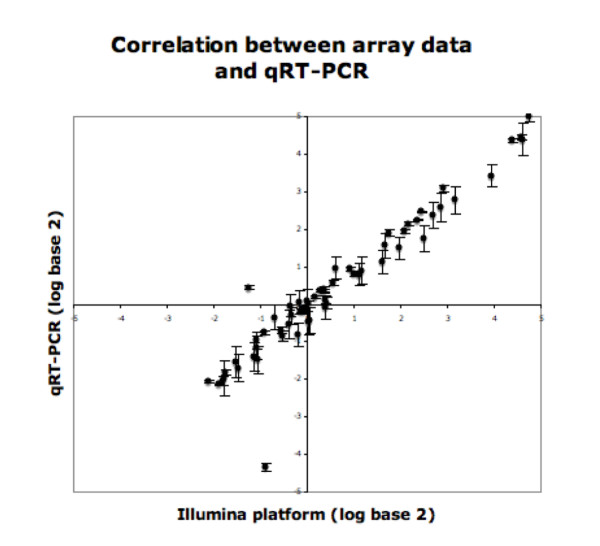
**qRT-PCR validation of miRNA profiling**. Comparison of log base 2 array results to qRT-PCR for 10 miRNAs in 5 tumors and 2 normals. All qRT-PCR values were normalized to U6 snRNA. Error bars indicate the standard error associated with triplicate qRT-PCR results. Pearson Correlation r between the data generated by qRT-PCR and the data generated by array was 0.95.

Array data was validated by by qRT-PCR for 10 miRNAs (mir-1, miR-10b, miR-135b, miR-147, miR-31, miR-33, miR-503, miR-552, miR-592, miR-622). This analysis was performed using RNA from 5 tumor and 2 normal samples (7 samples total). miRNA expression levels measured by qRT-PCR showed remarkably similar expression levels to those obtained by Illumina arrays (Figure 4). Data generated by these two different methodologies were highly correlated (Pearson Correlation r = 0.95).

### Predicted Targets for miRNAs Significantly Altered in Colon Cancer are Enriched in Cancer-related Signaling Pathways

**Figure 5:**
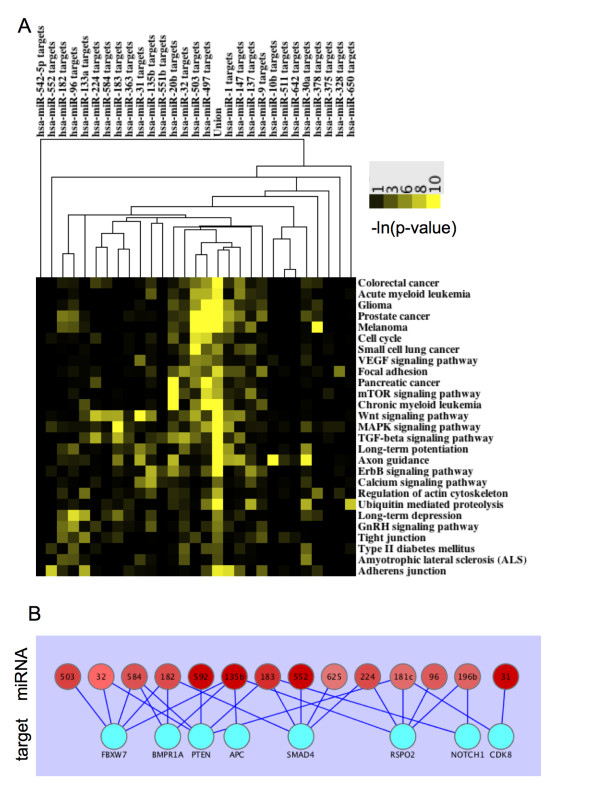
**Functional analyses of miRNAs altered in colon cancer**. (A) Kegg Pathways enriched in the predicted targets of miRNAs perturbed in CC. The results of the analyses were converted into a heatmap using the -ln (p-value). (B) Predicted interactions between miRNAs up-regulated in CC and known CC drivers.

The Diana miRpath application [32] was used to define pathways enriched in the predicted targets for CC significant miRNAs. miRpath compiles lists of predicted miRNA targets and looks for target enrichment in the KEGG pathway database. The results of the analyses were converted into a heat map using the -ln(p-value) and were clustered on the pathway axis and the miRNA putative target axis. The 174 pathways were filtered down to 27 by additionally requiring the miRNA putative targets to be enriched ((-Ln [p-value]) > 3) in at least 4 of the different pathways. The results of this analysis is that many pathways describing specific types of tumors, or signaling pathways involving oncogenesis show enrichment in the predicted target lists generated from miRNAs found to be altered in colon tumors (Figure 5A).

### Induced miRNAs are predicted to Target Tumor Suppressors

Recently, the results of a forward genetic screen for tumor suppressors in mice independently validated the role of a number of genes in intestinal tumorigenesis [30]. Therefore, we sought to determine whether miRNAs up-regulated in colon tumors were predicted to interact with the 3' UTR of these tumor suppressors, potentially attenuating their transcript level/translation status. Of the 15 genes described as "drivers" of CC [30], interactions were found between eight genes and 8 miRNAs reported to be up-regulated in colon tumors in the current study. Furthermore, in four of these cases, five separate up-regulated miRNAs were predicted to bind to the 3' UTR of the tumor suppressor (Figure 5B).

### Similarities in Colon Cancer and Stem Cell Differentiation

**Figure 6:**
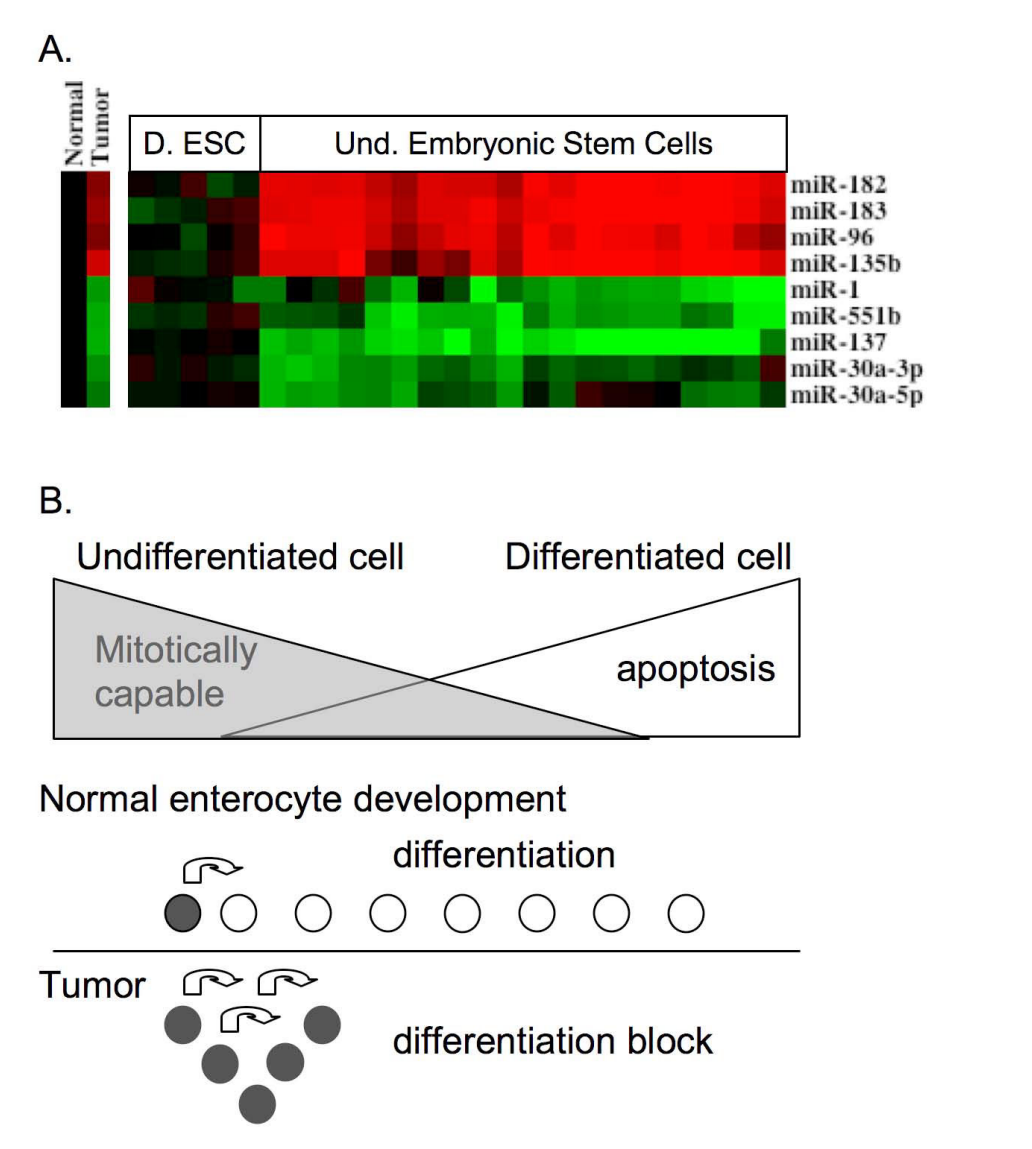
**Colon Cancers show partial reactivation of embryonic stem cell miRNA expression patterns**. (A) Expression patterns of colon cancer miRNAs showing similar changes between differentiated and undifferentiated embryonic stem cells. Stem cell miRNA expression data is shown relative to the average value of the differentiated stem cells. (B) Proposed model for intestinal tumorigenesis.

The differentially expressed colon tumor miRNA set was also looked at in stem cell-related miRNA profiles, previously published using the Illumina platform (Figure 6A) [31]. Of interest was the expression profile of the miR-96, miR-182 and miR-183 clusters as well as miR-135b, which were up-regulated in embryonic stem cells and whose expression decreased following differentiation. Similarly, miR-1, miR-551b, miR-137, miR-30a-3p and miR-30a-5p were all expressed at lower levels in embryonic stem cells relative to differentiated cells. This is consistent with the down-regulation we report for these miRNAs in colon tumors relative to normal tissue samples.

## Discussion

This study has revealed that a number of miRNAs are strongly differentially expressed during the development of CC, including miRNAs not previously reported. Using the Illumina platform, 39 miRNAs were identified that show highly statistically significant and meaningful fold change alterations in CC tumors. The core of this response showed very similar patterns in all colon tumor types studied. In addition, six miRNAs were identified that are significantly differentially expressed in dMMR tumors compared to pMMR tumors.

As previously described, high-level correlations in the miRNA data derived from both technical and biological replicate experiments were observed over a wide range of detection [21,33]. In addition, the impact of 2-fold fluctuations in the input RNA population was less than the impact of biological variability between normal samples. As expected, therefore, distinct expression signatures were observed in the current study between normal and colon tumor tissues. Furthermore, unsupervised hierarchical clustering and SVM results showed that this was robust and highly predictive in nature.

Several groups have published miRNA profiles of colon tumors using different platforms with or without an amplification step: RT-PCR for 150 miRNAs [17], microRNA microarray for 389 miRNAs [18], and LNA-based oligonucleotide arrays for ~315 miRNAs [19]. The present study is thus the most comprehensive, evaluating 735 miRNAs, the most statistically rigorous, using a Bonferroni correction to the multiple testing problem requiring large fold changes and used a macrodissected set of tumor tissues, therefore minimizing the effect from non-malignant cells. Most notably, 18 of the 39 miRNAs we found significantly altered in CC, have been previously reported by systematic RT-PCR [17].

Previous work with spotted arrays and qRT-PCR revealed the induction of miR-21 [17,19] and the repression of miR-143 [20] in CC compared to normal colon samples. Surprisingly, these miRNA did not show differential expression on the Illumina platform. We conducted separate qRT-PCR for these miRNAs and were able to see induction of miR-21 and repression of miR-143 in our tumor set compared to normal tissue. miR-21 showed a significant P-value (P = 2.436e-5 but had an average fold change of only 1.02 between groups. Non-responsive probes for miR-21 and miR-143 all had very high expression levels and very low variance, while responsive probes had higher variance and lower average values.

In order to understand the rationale for this discrepancy, we plotted the miRNA expression profiles relative to the average of normal tissue, alongside the absolute raw expression levels for each probe [see Additional file 6]. Our results demonstrate that miRNAs with high raw intensity values as well as a negative control, show a tight distribution of values whereas that distribution is much more variable in responsive probes. Further comparison reveals that non-responsive probes have very high expression levels and very low variance, while responsive probes have higher variance and lower average values. These effects are also evident following normalization. Filtering the expression dataset for probes with high average expression and low variance reveals 28 probes with these features [see Additional file 7].

Our observation of high expression "silent" probes is consistent with a probe cDNA hybridization model where all probe-binding sites are occupied. According to this model, further increases or decreases in miRNA levels will not be visible by array analyses due to binding saturation. This creates a ceiling above which any change, either increase or decrease in miRNA level, will remain undetectable. Additional evidence in support of this model can be found by looking at the relative concentrations of these miRNA in deep sequencing. As an example, in some cases miR-21 made up 50% of the pool in deep sequencing [21].

Comparison of arrays generated with both the Illumina platform and spotted arrays for 4 different commonly used cell lines showed that following removal of high average expression low variance "silent" Illumina probes, and low signal probes from the cDNA arrays, the remaining probes cluster together independently of the array platform [see Additional File 8]. This information coupled with our qRT-PCR verification of responsive miRNA, indicated that the Illumina arrays have very low false positive rates; but are potentially susceptible to false negative signal events.

dMMR and pMMR tumors showed differential expression of a small set of miRNA. The molecular etiology of those tumors involving dMMR is very heterogeneous, involving several different genes and numerous mechanisms of gene inactivation, including epigenetic, somatic and germline alterations. Among sporadic CC, the vast majority of cases with dMMR are due to inactivation of *MLH1 *(~95%), with *MSH2 *and *MSH6 *accounting for ~5% and < 1%, respectively [34]. For *MLH1*, the most common mechanism (~90% of cases) of gene inactivation is promoter hypermethylation [35]. In this study, analyses of dMMR cases were specifically confined to those with loss of *MLH1 *due to promoter hypermethylation. This was done to achieve a homogeneous group. Thus, the results derived from this study primarily reflect the biology of sporadic *MLH1 *CC.

Furthermore, *hMLH1 *methylation-associated microsatellite instability has also been strongly associated with tumors that express the CpG Island Methylator Phenotype (CIMP) [36]. As *hMLH1 *methylation-associated microsatellite instability generally does not occur among sporadic cases outside the context of CIMP, it appears that the underlying basis for mismatch repair deficiency among this select group of sporadic colon cancer is a broader epigenetic control defect that affects *hMLH1 *in some, but not all CIMP tumors. CIMP tumors represent another subset of all CC. Thus, the few pMMR samples that closely resemble the dMMR subset as a group, with respect to the miRNA profile, may have an underlying CIMP phenotype, which would be common to both groups. In fact, all but one of the cases, for which the CIMP phenotype was available, cluster within a single group containing both dMMR and pMMR.

Collectively, our data supports a model for colon tumorigenesis encompassing miRNA::mRNA interactions. In support of this model, the predicted mRNA target lists compiled from the 39 altered miRNAs in CC are enriched in tumorigenesis and cancer-related signaling pathways. The simplest explanation of this phenomenon is that up-regulated miRNAs directly or indirectly decrease expression of tumor suppressor proteins in contrast to down-regulated miRNAs that may lead to increased oncogene expression.

The predicted interactions were further explored between up-regulated miRNAs and a set of "drivers" of CC as defined by a forward genetic screen in mice [30]. Many of the induced miRNA found in this study were predicted to interact with the tumor suppressors. In addition, *PTEN *[37], *SMAD4 *[38], and *NOTCH1 *[39], are known tumor suppressors whose transcript or protein levels are decreased in tumors. miRNA-mediated decreases in tumor suppressors provide an elegant explanation for the observed tumor expression patterns.

In this analysis, highly significant increases were observed in miR-135b in CC and the interaction between miR-135b and *APC *was identified as relevant. It has recently been shown that increased levels of miR-135a/b lead directly to decreased protein expression of the CC tumor suppressor *APC *via a direct binding interaction between miR-135b and *APC *mRNA 3' UTR [40]. Although *APC *mutations are found in a majority of CC, deregulation of miR-135b may have an adverse effect on *APC *in the remainder of cases.

Inadvertent expression and activation of tumor suppressors could easily disrupt normal growth. High levels of intestinal cell proliferation are required to offset the very high turnover rate of intestinal tissue [41]. We propose that miRNA mediated attenuation of transcription/translation of tumor suppressors is a necessary step in normal intestinal development allowing for cell proliferation. With differentiation, the intestinal epithelial cells no longer replicate, which coincides with a change in miRNA expression and increased levels of tumor suppressors. miRNA expression profiles found in CC show striking similarities with those miRNA profiles found in undifferentiated embryonic stem cells relative to differentiated stem cells (Figure 6A) and cancer cell lines [31]. Embryonic stem cells and colon tumor cells are both capable of almost unlimited mitotic division. A potential interpretation of this observation is that the intestinal crypt cells fail to properly differentiate and may instead continue to actively divide leading to tumor formation (Figure 6B). This is further supported by the observed decreases in miR-1 and miR-133a, which are involved in maintaining the differentiation status of muscle cells [42]. In addition, miR-1 over-expression has been shown to cause expression of differentiated muscle cell mRNA [43]. The decreased levels of miR-1 and miR-133a in dMMR tumors relative to pMMR tumors may also explain why sporadic CC with dMMR show poor differentiation.

Temporal patterns of gene expression during cell-cell adhesion-initiated polarization of cultured human Caco-2 cells found similar transcript changes to those observed during migration and differentiation of intestinal epithelial cells *in vivo*, despite the absence of morphogen gradients and interactions with stromal cells characteristic of enterocyte differentiation *in situ *[44]. Here, we propose that miRNAs may be involved in these changes and that the miRNA profile within each cell is modified as cells stop dividing, undergo chromatin remodeling and become further differentiated with limited ability to replicate.

Taken together, these findings suggest that the inability to properly differentiate due to loss of epigenetic control may be an important factor in the development of colon cancer. The role of induced miRNA found in tumors may be to (i) drive tumorigenesis by attenuating the translation of tumor suppressors thereby maintaining a state capable of cell division; or (ii) act as passengers whose levels reflect that they are locked into a state of uncontrolled cell replication.

Several basic questions arise from these findings. How and why are miRNA expression levels altered and how can they be modified? Our analyses show that miR-135b was highly up-regulated in colon tumors. *PDRM5 *is a tumor suppressor and a target of epigenetic silencing in CC [45]. Of interest, *PDRM5 *has been shown to bind to the promoter of miR-135b and *PDRM5 *silencing leads to increased levels of miR-135b. Mechanistically, *PRDM5 *recruits *HDAC1 *and *G9a *to the miR-135b promoter, and this chromatin remodeling results in decreased miR-135b expression. Decreases in *PDRM5 *expression levels lead to increased expression of miR-135b presumably due to loss of recruitment of this chromatin-remodeling complex. Intriguingly, *PDRM5 *was also found by CHIP-CHIP to be present at the promoter regions of miR-9, miR-378, miR-196b, miR-96-182-183, as well as additional colon cancer relevant miRNA miR-21 and miR-143-145 [46]. Decreases in *PDRM5 *were associated with both increases and decreases in miRNA expression. miR-9 was also down-regulated in invasive breast cancers via promoter hypermethylation [47]. Therefore, *PDRM5*-mediated chromatin remodeling may be a general epigenetic mechanism related to a wide range of tumors. Drugs that modify chromatin structure and methylation status in a sequence specific fashion may be useful in colon cancer treatment due to the reversible nature of histone modification and DNA methylation. Those that modify miRNA transcript level or mimic/inhibit miRNA function may also be useful.

## Conclusion

The data presented here unifies miRNA expression platforms, broadens the miRNAome associated with CC and defines highly significant differences in miRNA expression between dMMR and pMMR tumors. We hypothesize that the differential miRNA expression patterns observed may modify oncogene and tumor suppressor protein expression and thereby may promote tumorigenesis by blocking the proper differentiation of intestinal progenitor cells, consequently leading to improper cell division and tumor formation. By identifying the miRNA alterations associated with CC at a high level of definition in a statistically powerful dataset, we have provided an important template on which to discover critical biomarkers and develop more effective treatment protocols for colon cancer at all stages.

## Competing interests

The authors declare that they have no competing interests.

## Authors' contributions

CJS and SNT conceived the project. ALS, AJF, ALO, KATS, BWM, LAB, SS, LW, TCS, SNT and CJS were involved in experimental design. AJF carried out macro-dissection and MMR determination. PMB carried out RNA extraction. JMC carried out the miRNA profiling. VT carried out qRT-PCR. ALS, ALO, KATS, BWM, SMR conducted analyses of expression data. ALS drafted the manuscript and carried out cross data set comparisons. AJF, PMB, SS, CR, SNT and CJS contributed to the manuscript. All authors read and approved the final manuscript.

## Pre-publication history

The pre-publication history for this paper can be accessed here:

http://www.biomedcentral.com/1471-2407/9/401/prepub

## Supplementary Material

Additional file 1 **qRT-PCR primer sequences**. Sequences used for qRT-PCR of miRNA levels. Click here for file

Additional file 2 **Tissue clinical data**. Additional clinical data shown in Table 1 broken down by tumor id. Click here for file

Additional file 3 **miRNA profiles in Colon Cancer and normal tissues**. All data, both raw and normalized. A)Raw Log base 2 transformed miRNA data B)Quantile normalized log base 2 miRNA data C) Averaged log base 2 quantile normalized miRNA data D) Averaged, quantile normalized miRNA data E)quantile normalized averaged data normalized by average of normal colon tissue. Click here for file

Additional file 4 **SVM confusion matrices**. Confusion matrices for A) normal vs tumor comparison using all miRNA B) dMMR vs pMMR tumor comparison. Click here for file

Additional file 5 **Complete statistical data**. A linear mixed effects model was used to determine statistically significant miRNAs for A) normal tissue compared with colon cancer tissue and B) dMMR tumor tissue compared with pMMR tumor tissue. Click here for file

Additional file 6 **Illumina platform analyses**. Illumina platform performance analysis for previously reported miRNAs altered in CC. Heat map describing fold change from normal tissue for known high concentration miRNAs (let-7a), a negative control element (neg.con.), miRNAs reported involved in colon tumorigenesis (miR-21 and miR-143) and miRNAs we observed to be altered in tumor specimens (miR-31, miR-135b, miR-30a-3p). The log base 2 raw fluorescent intensity is shown for each miRNA. The highest value found on the array was less than 16 on the log base 2 scale. Click here for file

Additional file 7 **Probes with high average expression and low variance**. Probes with high average expression and low variance. Click here for file

Additional file 8 **Unsupervised hierarchical clustering of cell line miRNA profiles obtained from Illumina platform and cDNA arrays**. Following removal of high expressing low variability Illumina probes and low expressing cDNA probes the remaining miRNA cluster together by cell line of sample origin. Click here for file
