# Supplementary figures and images for: Human colon cancer profiles show differential microRNA expression depending on mismatch repair status and are characteristic of undifferentiated proliferative states
Source: BMC Cancer. 2009 Nov 18;9:401. doi: 10.1186/1471-2407-9-401 (PMC2787532; doi:10.1186/1471-2407-9-401)

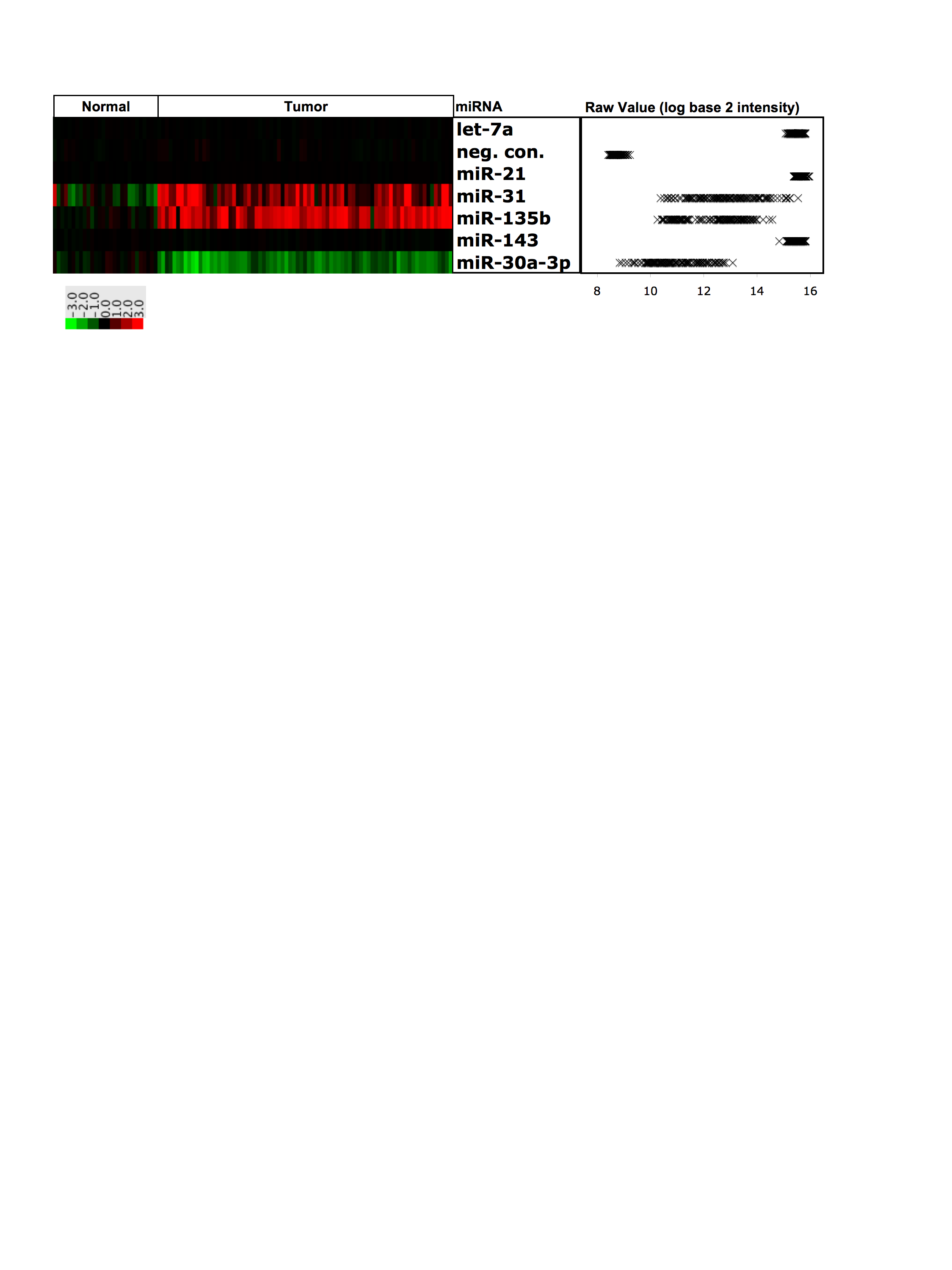

Supplement: Additional file 6 — Illumina platform analyses. Illumina platform performance analysis for previously reported miRNAs altered in CC. Heat map describing fold change from normal tissue for known high concentration miRNAs (let-7a), a negative control element (neg.con.), miRNAs reported involved in colon tumorigenesis (miR-21 and miR-143) and miRNAs we observed to be altered in tumor specimens (miR-31, miR-135b, miR-30a-3p). The log base 2 raw fluorescent intensity is shown for each miRNA. The highest value found on the array was less than 16 on the log base 2 scale. [file 1471-2407-9-401-S6.PNG]

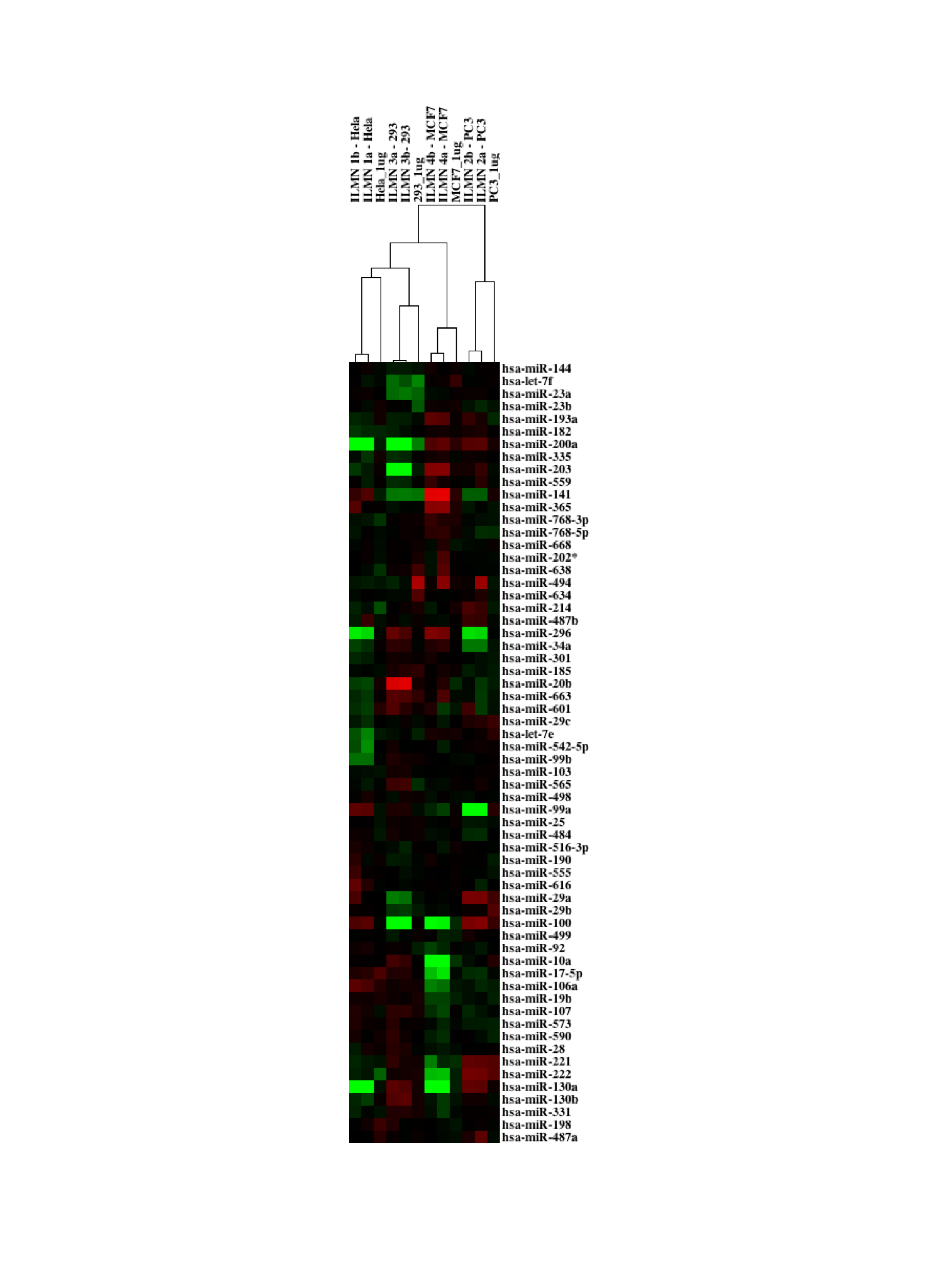

Supplement: Additional file 8 — Unsupervised hierarchical clustering of cell line miRNA profiles obtained from Illumina platform and cDNA arrays. Following removal of high expressing low variability Illumina probes and low expressing cDNA probes the remaining miRNA cluster together by cell line of sample origin. [file 1471-2407-9-401-S8.PNG]
